# Histological and Immunohistochemical Characteristics for Hereditary Breast Cancer Risk in a Cohort of Brazilian Women

**DOI:** 10.1055/s-0042-1743103

**Published:** 2022-04-25

**Authors:** Renata Mendes de Freitas, Maximiliano Ribeiro Guerra, Vívian Assis Fayer, Angélica Atala Lombelo Campos, Jane Rocha Duarte Cintra, Joan Warren, Rafaela Russi Ervilha, Camila Damasceno de Paula, Maria Teresa Bustamante-Teixeira

**Affiliations:** 1 Department of Public Health, Faculdade de Medicina, Universidade Federal de Juiz de Fora, Juiz de Fora, MG, Brazil; 2 Epidemiology of Congenital Malformations Laboratory, Fundação Oswaldo Cruz, Rio de Janeiro, RJ, Brazil; 3 Oncology Institute, Hospital 9 de Julho, Juiz de Fora, MG, Brazil; 4 Independent Researcher, Washington, United States

**Keywords:** breast cancer, hereditary breast and ovarian cancer syndrome, cohort studies, immunohistochemistry, genetic counseling, câncer de mama, síndrome hereditária de câncer de mama e ovário, estudos de coorte, imuno-histoquímica, aconselhamento genético

## Abstract

**Objective** The study aimed to characterize the clinical, histological, and immunohistochemical profile of women with invasive breast cancer, according to the risk for Hereditary Predisposition Breast and Ovarian Cancer Syndrome in a Brazilian population.

**Methods** This is a retrospective study performed from a hospital-based cohort of 522 women, diagnosed with breast cancer treated at an oncology referral center in the Southeast region of Brazil, between 2014 and 2016.

**Results** Among the 430 women diagnosed with invasive breast cancer who composed the study population, 127 (29.5%) were classified as at increased risk for hereditary predisposition to breast and ovarian cancer syndrome. There was a lower level of education in patients at increased risk (34.6%) when compared with those at usual risk (46.0%). Regarding tumor characteristics, women at increased risk had higher percentages of the disease diagnosed at an advanced stage (32.3%), and with tumors > 2cm (63.0%), with increased prevalence for both characteristics, when compared with those at usual risk. Furthermore, we found higher percentages of HG3 (43.3%) and K _i_ -67 ≥ 25% (64.6%) in women at increased risk, with prevalence being about twice as high in this group. The presence of triple-negative tumors was observed as 25.2% in women at increased risk and 6.0% in women at usual risk, with the prevalence of absence of biomarkers being 2.5 times higher among women in the increased risk group.

**Conclusion** From the clinical criteria routinely used in the diagnosis of breast cancer, the care practice of genetic counseling for patients at increased risk of hereditary breast cancer in contexts such as Brazil is still scarce.

## Resumo

**Objetivo** O presente estudo buscou caracterizar o perfil clínico, histológico e imunohistoquímico de mulheres com câncer de mama invasivo segundo o risco para a Síndrome de Predisposição Hereditária ao Câncer de Mama e Ovário em uma população brasileira.

**Métodos** Trata-se de um estudo retrospectivo realizado a partir de uma coorte hospitalar composta por 522 mulheres diagnosticadas com câncer de mama entre 2014 e 2016 assistidas em um centro de referência oncológica localizado na região sudeste brasileira.

**Resultados** Entre as 430 mulheres diagnosticadas com câncer de mama invasivo que compuseram a população de estudo, 127 (29,5%) foram classificadas como de risco aumentado para a síndrome de predisposição hereditária ao câncer de mama e ovário. Verificou-se menor nível de escolaridade nas pacientes com risco aumentado (34,6%) quando comparadas àquelas consideradas como de risco habitual (46,0%). Quanto às características do tumor, as mulheres de risco aumentado apresentaram maiores percentuais de doença diagnosticada em estádio avançado (32,3%) e com tumores > 2cm (63,0%), com prevalência aumentada para ambas as características, quando comparadas àquelas de risco habitual. Ainda nas mulheres de risco aumentado, foram encontrados maiores percentuais de GH3 (43,3%) e K _i_ -67 ≥ 25% (64,6%), com prevalência cerca de duas vezes maior neste grupo. A presença de tumores triplo-negativos foi observada em 25,2% nas mulheres de risco aumentado e 6,0% nas mulheres de risco habitual, com prevalência de ausência de biomarcadores 2,5 vezes maior entre as mulheres do grupo de risco aumentado.

**Conclusão** A partir dos critérios clínicos rotineiramente utilizados no diagnóstico do câncer de mama, a prática assistencial do aconselhamento genético para as pacientes com risco aumentado de câncer de mama hereditário em contextos como o do Brasil ainda é escarça.

## Introduction

Breast cancer has a multifactorial etiology associated with hormonal, reproductive, genetics, lifestyle-related factors, and it is more frequent in post-climacteric women. 1 Most of these tumors originate in the ductal epithelium and acquire an invasive capacity. However, other histological types are found due to the great heterogeneity and different carcinogenic profiles of the tumors. 2

Molecular biology has allowed the investigation of several genes associated with carcinogenesis, including the analysis of gene expression profiles of breast cancers, which makes it possible to correlate them with disease prognosis and with response to treatment. 3 In clinical practice, the immunohistochemical technique enables a quick, simple, and low-cost analysis of the expression of proteins that compose the tumor; additionally, it can evaluate the tumor grade and identify the molecular subtypes of breast cancer. 4

Some characteristics of the breast tumor are essential for the clinical follow-up of the patients. The cell proliferation marker K _i_ -67 has an increased expression in breast tumors that may be associated with a higher risk of recurrence and worse prognosis. 5 The histological grade given by the Nottingham Classification System refers to the sum of the tubular grade, nuclear grade, and mitotic index scores, indicating the degree of differentiation of the tumor tissue, which also influences the prognosis. Furthermore, it is noteworthy that the size of the tumor is related to the probability of recurrence and lymph node involvement. 4

The hereditary condition is the cause identified in 10 to 25% of breast and ovarian cancers, involving mutations in genes of high and moderate penetrance, such as the *BRCA1* and *BRCA2* genes. 6 The National Comprehensive Cancer Network (NCCN) defines criteria that help identify women prone to hereditary breast cancer. These criteria take into account the clinical manifestation of pre-climacteric ages and more aggressive breast carcinogenesis, with a prevalence of bilaterality, triple-negative subtype tumors diagnosed up to 60 years old, Ashkenazi Jewish ancestry, and an association with other malignant neoplasms that affect family members, such as ovarian, endometrial, pancreas, bowel, prostate, and male breast cancer. 7 8 9

Furthermore, the NCCN (2020) 9 and other medical organizations suggest potential candidates to specialists that would benefit from genetic testing and counseling, based mainly on personal and family history of cancer. 8 9 10 Family history is an important means used to identify individuals at risk of hereditary cancer; however, recommendations based on family history for genetic screening may be limited due to a poor family history, limited to first-degree relatives or to information inconsistently documented by professionals. 7 9 10 11

Therefore, frequent reviews of the recommendation guidelines to assess Hereditary Breast and Ovarian Cancer (HBOC) syndrome have been performed to advance the diagnosis and management of women with HBOC syndrome, which has enabled the incorporation of new and more comprehensive criteria. 6 10

In Brazil, there are few studies on the genetic profile of patients and family members at risk for HBOC syndrome. 12 This study seeks to characterize the clinical, histological, and immunohistochemical profile of women with invasive breast cancer, according to the risk for HBOC syndrome in a Brazilian population.

## Methods

This is a retrospective study, performed from a hospital-based cohort, consisting of 522 women diagnosed with breast cancer between 2014 and 2016, and treated at an oncology referral center in the Zona da Mata of Minas Gerais, in the southeastern region in Brazil.

Sociodemographic, clinic, and pathological data were extracted from medical records, and additional information was obtained through interviews with patients, as well as from analysis of pathological anatomy and immunohistochemical test results.

Through the criteria used to assess hereditary breast cancer risk, recommended by the NCCN (2020), 9 women were classified into two categories: increased and usual risk for hereditary breast cancer. The group with increased risk for hereditary breast cancer considered the presence of at least one of the clinical criteria for HBOC Syndrome, such as: age at diagnosis ≤ 45-years-old; triple-negative subtype diagnosed in women aged ≤ 60 years; diagnosis of breast cancer between 46–50-years-old, with at least one first or second-degree relative with malignant neoplasm in the breast or ovary; and a personal history of breast cancer with the presence of secondary malignant tumor in the same organ. 9 The group with usual risk for hereditary breast cancer was considered as the same found in the asymptomatic female population, which has environmental and hormonal factors as the main risk conditions for the development of the disease.

The study excluded women with in situ breast cancer ( *n* = 42) and those without information about at least one of the biomarkers of the tumor for estrogen, progesterone, and HER-2 ( *n* = 50).

The characterization of the pathological profile of breast carcinoma was performed using the following variables: stage at diagnosis (early – I, intermediate – II, advanced stage – III and IV), histological type (ductal, lobular, others), tumor size (≤ 2 cm, > 2 cm), lymph node involvement, histological grade (HG1–well differentiated, HG2–moderately differentiated, HG3–poorly differentiated), K _i_ -67 cell proliferation index (< 25%, ≥ 25%). Considering the relationship between the increased expression of K _i_ -67 and HG3, according to Gong et al. 5 and Delpech et al. 13 perineural invasion, vascular invasion, inflammatory infiltrate, multifocality, multicentricity, intraductal component, estrogen and progesterone hormone receptors (HR), HER-2 receptor, and immunohistochemical biomarkers (present: HR ± and HER-2+ or HR+ and HER-2-; absent: HR- and HER-2). And considering the presence of HER-2- only in tumors reported as 2+ or 3+ with confirmation by in situ hybridization technique. 14

From analyses stratified according to risk for hereditary breast cancer, the mean and respective 95% confidence interval (95% CI) for age at diagnosis, as well as absolute numbers and percentages for categorical variables were presented. The difference in the distribution of categorical variables according to the risk for hereditary breast cancer was assessed using the chi-square test (χ ^2^ ), and the significance level considered was 5%. For these variables were estimated the prevalence ratios (PR) and the respective 95% confidence interval (95% CI). The analysis was performed using the STATA (StataCorp. College Station, TX, USA) software, version 16.0.

The study was approved by the Research Ethics Committee of Federal University of Juiz de Fora (CEP/UFJF), CAAE: 5342919.0.0000.5147.

## Results

Among the 430 women diagnosed with invasive breast cancer who composed our study population, 127 (29.5%) were classified as at increased risk for HBOC Syndrome, according to the criteria recommended by the NCCN (2020). 9 For women at increased risk, the mean age was 42 years (95% CI: 40.9–44.0), and for those with usual risk, it was 63 years (95% CI: 61.9–64.2).

The majority of women were white (72.3%), with more than eight years of education (55.8%), and users of the public health service (60.5%). There was a lower level of education in patients at increased risk (36.4%) when compared with those considered to be at usual risk (46.0%) (PR = 0.70; 95% CI: 0.52–0.96) ( Table 1 ).

**Table 1:** Sociodemographic characteristics and family history of cancer according to the risk of predisposition for Hereditary Breast-Ovarian Cancer Syndrome (HBOC) in women treated

Variables			Usual risk		Increased risk			PR b (95% CI)
	n *	%	n	%	n	%	P a	
Age at diagnosis								
≥ 50 years old	299	69.5	282	93.1	17	13.4	0.000	1.00
< 50 years old	131	30.5	21	6.9	110	86.6		14.8 (9.3–23.6)
Skin color								
White	311	72.3	226	74.6	85	66.9	0.16	1.00
Non-white	113	26.3	72	23.8	41	32.3		1.33 (0.98–1.80)
Education (in years)								
> 8	240	55.8	158	52.0	82	64.6	0.05	1.00
≤ 8	183	42.6	139	46.0	44	34.6		0.70 (0.52–0.96)
Health service								
Private	170	39.5	124	40.9	46	36.2	0.40	1.00
Public	260	60.5	179	59.1	81	63.8		1.15 (0.85–1.56)
Family history of breast cancer								
Absent	299	69.5	222	73.3	77	60.6	0.01	1.00
Present	131	30.5	81	26.7	50	39.4		1.48 (1.11–1.98)
First-degree relative with breast cancer c								
Absent	368	85.6	267	88.0	101	79.5	0.02	1.00
Present	62	14.4	36	12.0	26	20.5		1.53 (1.09–2.14)

Abbreviations 95% CI, 95% confidence interval; PR, prevalence ratios.

Notes:

a chi-square (X ^2^ ) test, *p* < 0.05

b prevalence ratio calculated only for valid data

c first-degree relatives: parents, children, and siblings of the patient

* the difference in totals (N) is due to incompleteness of information.

Most of the investigated patients did not mention a history of breast cancer in the family (69.5%). However, for women at increased risk, 39.4% had a positive history of cancer in up to third-degree relative. When we considered only first-degree relatives with breast cancer in the group of women at increased risk (20.5%), a 50% higher prevalence was found in the increased risk group than in the usual risk group (PR = 1.5; 95% CI: 1.09–2.14). Regarding anatomopathological characteristics of the population of the study, it was observed that 74.4% had early-stage/intermediate type, 82.3% had invasive ductal histological type, tumor size > 2 cm (54.2%), non-involved lymph nodes (50.9%), histological grade 2 (37.7%), and K _i_ -67 < 25% (48.1%) ( Table 2 ).

**Table 2:** Histological and immunohistochemical characteristics of the breast tumor, according to the risk of predisposition for Hereditary Breast-Ovarian Cancer Syndrome (HBOC) in women treated

Variables			Usual risk		Increased risk			
	n *	%	n	%	n	%	P a	PR b (95% CI)
Stage at diagnosis c								
Early stage/intermediate	320	74.4	234	77.2	86	67.7	0.04	1.00
Advanced	110	25.6	69	22.8	41	32.3		1.39 (1.02–1.88)
Histological type								
Invasive lobular	34	7.9	27	9.0	7	5.5	0.51	1.00
Others	40	9.3	28	9.2	12	9.5		1.46 (0.65–3.28)
Invasive ductal	354	82.3	246	81.2	108	85.0		1.48 (0.75–2.92)
Tumor size								
≤ 2cm	195	45.3	148	48.8	47	37.0	0.04	1.00
> 2cm	233	54.2	153	50.5	80	63.0		1.42 (1.05–1.93)
Compromised lymph nodes								
No	219	50.9	163	53.8	56	44.0	0.07	1.00
Yes	211	49.1	140	46.2	71	56.0		1.32 (0.98–1.77)
Histological grade (HG) d								
HG 1	90	20.9	75	24.8	15	11.8	<0.01	1.00
HG 2	162	37.7	115	38.0	47	37.0		1.74 (1.03–2.93)
HG 3	125	29.1	70	23.0	55	43.3		2.64 (1.60–4.36)
Ki67								
< 25%	207	48.1	166	54.8	41	32.3	<0.01	1.00
≥ 25%	204	47.5	122	40.3	82	64.6		2.03 (1.47–2.80)
Perineural invasion								
Absent	388	90.2	271	89.4	117	92.0	0.39	1.00
Present	42	9.8	32	10.6	10	8.0		0.79 (0.45–1.38)
Vascular invasion								
Absent	334	77.7	234	77.2	100	78.7	0.73	1.00
Present	96	22.3	69	22.8	27	21.3		0.94 (0.66–1.35)
Inflammatory infiltrate								
Absent	319	74.2	229	75.6	90	71.0	0.31	1.00
Present	111	25.8	74	24.4	37	29.0		1.18 (0.86–1.62)
Multifocality								
Absent	387	90.0	276	91.1	111	87.4	0.25	1.00
Present	43	10.0	27	8.9	16	12.6		1.30 (0.85–1.97)
Multicentricity								
Absent	416	96.7	295	97.4	121	95.3	0.27	1.00
Present	14	3.3	8	2.6	6	4.7		1.47 (0.79–2.75)
Intraductal component								
Absent	320	74.4	228	75.2	92	72.4	0.54	1.00
Present	110	25.6	75	24.8	35	27.6		1.11 (0.80–1.53)
Hormone receptors								
Present	357	83.0	268	88.4	89	70.0	<0.01	1.00
Absent	71	16.5	33	11.0	38	30.0		2.15 (1.62–2.85)
HER-2								
Present	74	17.2	51	16.8	23	18.1	0.55	1.00
Absent	348	80.9	245	80.9	103	81.1		1.05 (0.7–1.53)
Biomarkers e								
Present	380	88.4	285	94.0	95	74.8	<0.01	1.00
Absent	50	11.6	18	6.0	32	25.2		2.56 (1.95–3.36)

Abbreviations 95% CI, 95% confidence interval; PR, prevalence ratios. Notes:

a chi-square (X ^2^ ) test, *p* < 0.05.

b Prevalence ratio calculated only for valid data.

c Stage at diagnosis: early stage (I); intermediate (II); advanced (III and IV).

d Histological grade: HG1: well differentiated; HG2: moderately differentiated; HG3: poorly differentiated.

e Present biomarkers: HR ± and HER-2+ or HR+ and HER-2-; absent biomarkers: HR- and HER-2- (triple negative).

* The difference in totals (N) is due to incompleteness of information.

There was a significant difference between the groups regarding staging, tumor size, histological grade, and K _i_ -67 index. Women at increased risk had higher percentages of diagnosed disease at an advanced stage (32.3%), with tumors > 2cm (63.0%), and an increased prevalence (∼1.4 times) for both characteristics, when compared with those at usual risk.

Also in women at increased risk, higher percentages of HG3 (43.3%) and K _i_ -67 ≥ 25% (64.6%) were found, with a prevalence about twice as high in this group (PR = 2.64 and 2.03, respectively). On the other hand, in women at usual risk, higher percentage of more differentiated tumors was identified, with HG2 (38.0%) and K _i_ -67 < 25% being intermediate or low (54.8%).

There was no significant difference in the distribution of histological types between groups. The lymph node involvement was 56.0% in women at increased risk, and 46.2% in women at usual risk, although it was also statistically insignificant ( Table 2 ).

Regarding estrogen and/or progesterone hormone receptors (HR), an absence of HR expression was found in 30.0% of the women at increased risk, compared with 11.0% of women at usual risk ( *p* < 0.01), with a prevalence about two times higher in the increased risk group. Higher percentages of HER-2 expression negativity were also observed in both groups (increased risk: 81.8% vs. usual risk: 80.9%), with no significant difference between groups ( Table 2 ).

The biomarkers (HR and/or HER-2) used to classify breast cancer subtypes through the immunohistochemical technique had a percentage of 88.4 in the study population. However, its distribution was significantly different between the groups, with an absence of expression of biomarkers, that is, triple-negative tumors in 25.2% of women at increased risk, and 6.0% in women at usual risk, with a prevalence of absence of biomarkers 2.5 times higher among women in the increased risk group.

The other variables related to tumor characteristics did not show a significant difference in distribution according to the groups considered ( Table 2 ).

**Fig. 1:**
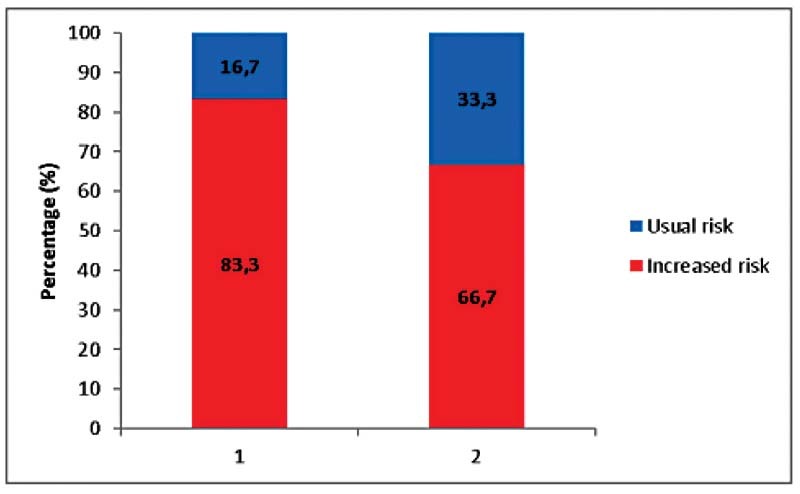
K _i_ -67 ≥ 25% with absent biomarkers (Column 1); and K _i_ -67 ≥ 25% and absent HR (Column 2) with simultaneous occurrence for each considered group.

Figure 1 shows the distribution of the tumors' histological and immunohistochemical characteristics with simultaneity of occurrence between the following variables: K _i_ -67 ≥ 25%, absents biomarkers, and absents HR for each group considered.

When considering K _i_ -67 ≥ 25% and absent biomarkers, a percentage of 83.3 versus 16.7% was observed in the increased and usual risk groups, respectively. Higher percentages were also observed in the increased risk group when K _i_ -67 ≥ 25% and absent HR (66.7%) were compared simultaneously to the usual risk group (33.3%).

## Discussion

Women in the increased risk for hereditary breast cancer group represented 29.5% of the study population and had a mean age of 42 years at diagnosis, which is far below the mean age of the group considered as usual risk (62 years), according to the results found in other studies. 15

Between 10 to 25% of breast and ovarian neoplasms are considered hereditary and will manifest earlier in women with some risk factors related to this heredity, that is, history cases of breast, ovarian, and male breast cancers due to the detection of some genetic alteration, especially in the *BRCA1* and *BRCA2* genes, which reinforces the benefit that these women would have when performing an improved screening. 6 7 10

In our study, the frequency of women at increased risk for clinical criteria for HBOC syndrome was higher than reported in the literature, which generally addresses Caucasian populations from Europe and the United States. Moreover, it should be noted that divergence of some criteria and guidelines between referral institutions can make it difficult to reach a consensus on the identification of eligible patients for investigation of hereditary cancer. 10

In Brazil, access to risk evaluation for hereditary cancers and genetic screenings is limited. Brazilian experts propose recommendations to expand early diagnosis, risk management, and treatment of hereditary breast cancer, as well as provide epidemiological information about the Brazilian population. The identification of women at increased risk for hereditary breast cancer allows patients and their physicians to assess the available options to mitigate the risk of developing breast cancer, including more frequent screening, chemoprophylaxis, and even prophylactic mastectomy. 6

For hereditary breast cancer, family history is the most accepted risk factor among the scientific community, with a risk increase of 2 to 4 times in the presence of family members affected by breast cancer, especially if they have been diagnosed at an early age. 7 10 16 In this study, family history—specifically the presence of affected first-degree relatives—was evaluated, observing a higher prevalence of these conditions in the group of women at increased risk. When genetic inheritance is strongly transferred between first-degree relatives, the relative risk of developing breast cancer in pre-climacteric women becomes higher. 16

Regarding sociodemographic characteristics, white skin color was the most prevalent among the women evaluated. However, there was a higher number of non-white women in the increased risk for hereditary breast cancer group. It is noteworthy that the worst prognostic features for breast cancer, such as younger age, late diagnosis, and triple-negative tumors, are more frequent in black women. 17

Nevertheless, the study performed by Fejerman et al. (2009) 18 showed that for every 25% increase in European ancestry, there was a 20% increase in the risk of breast cancer. It is known that the Brazilian population is mainly composed of European, African, and Amerindian ancestral roots, among others, presenting a high genomic diversity. 19 However, little is known about the profile of genetic ancestry in the Brazilian population related to breast cancer. In the only study with this approach in a Brazilian population identified in our literature review, Fernandes et al. (2016), 12 observed some trends or associations related to genetic ancestry with more aggressive cancer behavior, including higher histological grade in patients whose African component was greater. Nonetheless, this investigation was performed in a cohort that may not represent the diversity of the Brazilian population.

The higher education level predominant in the group of women at increased risk ( *p* = 0.05) may highlight the relationship between educational level and socioeconomic status—which also interferes with greater access to information about the risk factors that permeate the development of cancer—and access to preventive measures, particularly important in women with hereditary predisposition to breast cancer. 19 20

In addition to a family history of cancer, tumor characteristics can be of considerable importance in women at increased risk for hereditary breast cancer, where histopathological findings can be potentially useful in predicting the presence of germline mutation, along with the already mentioned criteria for predisposition to HBOC syndrome. 21 Furthermore, some characteristics of breast cancer are considered prognostic factors of the disease, including the histological type, tumor size, lymph node involvement, and histological grade. 4 They may also present histological and molecular differences that are related to sporadic and hereditary cancers, helping define characteristics that are more prevalent in women at risk for HBOC syndrome. 22 23 24

Studies show that tumors related to HBOC syndrome are often larger, less poorly differentiated, with high cell proliferation markers, predominance of the invasive ductal histological type, and triple-negative subtype, leading to a more aggressive form of the disease. 22 23 24 25 26 This study corroborates these results, as there was a higher prevalence of advanced ductal carcinomas, tumor size greater than 2 cm, lymph nodes involvement, poorly differentiated tumors, K _i_ -67 ≥ 25%, and triple-negative subtype in the increased risk group.

It is known that advanced stages influence the therapeutic options and prognosis of patients, and among the histological types, invasive ductal carcinoma is the most common, accounting for approximately 70% of all prevalent cases of breast cancer. 20 In this study, a higher prevalence of the invasive ductal histological type was also observed in both the increased risk and usual risk groups (85.0%; 81.2%, respectively). In general, women affected by invasive ductal carcinoma have greater lymphatic involvement, which was also observed in the study population in general. 21 22 23 24 25 26 27 However, there was no data regarding when the study population was stratified by risk of hereditary cancer.

The histological grade (HG) of the invasive carcinomas is an important feature and must be evaluated to guide the therapeutic approach and predict the prognosis. 28 In the increased risk group, the frequency of HG3 was higher ( *p* < 0.01), as well as K _i_ -67 ≥ 25% ( *p* < 0.01), compared with the usual risk group. The literature points to higher HG and K _i_ -67 values in women with hereditary predisposition. 5 29 30

In the study of Mavaddat et al. (2012), 21 performed by the Consortium of Investigators of Modifiers of *BRCA1* and *BRCA2* (CIMBA), HG3 tumors were identified in 77.0% and 50.0% of women with mutations in the *BRCA1* and *BRCA2* genes, respectively.

Newman (2015) 31 suggests an inverse correlation between low incidence and higher breast cancer mortality rates in African American women compared with white women, with a 67% higher risk of death due to breast cancer among African American women.

Regarding hormone receptors (HR), the higher frequency of cases with absence HR was observed in the increased risk for hereditary breast cancer group, compared with the usual risk group ( *p* < 0.01), which reinforces the premise that women at risk for hereditary predisposition are more prone to estrogen receptor negativity. 30 The presence of hormone receptors in tumor tissue is related to indicators of good prognosis, with a lower histological grade and lower rates of cell proliferation. 3 4 18

The higher prevalence of triple-negative subtypes tumors, characterized by the absence of hormone receptors and HER-2, in women at increased risk (PR = 2.56; 95% CI: 1.95–3.36) also corroborates findings in the scientific literature. In the meta-analysis performed by Tun et al. (2014), 32 it was observed that in a population with increased risk characteristics, women with triple-negative breast cancer are 5.7 times more likely to have a hereditary predisposition compared with the non-triple-negative immunophenotype. Fernandes et al. (2019) 33 performed the same analysis, and their results showed the presence of tumors with HG3 in 56.5% and 25.0% of women with mutations in the *BRCA1* and *BRCA2* genes, respectively, thus corroborating the higher frequency of this finding in women at risk of HBOC syndrome.

As observed in the study by Fernandes et al. (2019), 33 most cases of triple-negativity occur in women with hereditary mutations in the *BRCA1* and *BRCA2* genes (51.1%). Moreover, according to data presented by Young et al. (2009), 34 women with this subtype of breast cancer diagnosed in pre-climacteric age should be candidates of a risk assessment test for HBOC, even in the absence of a family history of breast and ovary cancer. Therefore, the NCCN guidelines recommend a genetic investigation for all triple-negative breast cancer aged ≤ 60 years. 9

When evaluating the simultaneous occurrence of some characteristics in the categorized groups, we sought to verify that the information routinely collected at the time of diagnosis could favor the identification of women at increased risk for HBOC. Higher percentages were observed in the increased risk group, when compared with those of the usual risk group, regarding the presence of K _i_ -67 ≥ 25% in triple-negative tumors (83.3% vs. 16.7%), K _i_ -67 ≥ 25% and absence of hormone receptors (66.7% vs. 33.3%). The data presented suggests that a high K _i_ -67 proliferation index with the absence of expression of biomarkers could be clinical indicators of increased risk for HBOC.

In a retrospective analysis performed by Liang et al. (2020), 35 the authors evaluated the interaction between K _i_ -67 and HG in the prognostic of different subtypes of breast cancer. The results indicated that K _i_ -67 expression was significantly associated with HG in all breast cancer patients, and that patients with increased K _i_ -67 or HG3 had reduced recurrence-free survival and a worse prognosis. 5 13 35

It is important to highlight that these characteristics alone provide relevant information at the time of the diagnosis. This was demonstrated in the study performed by Pérez-López et al. (2016), 36 when considering tumor size, lymph node involvement HG3, and independent prognostic factors. In the same study, the high K _i_ -67 score increased the risk of breast cancer mortality by 2.7 times. 36

Most of the Brazilian studies published so far regarding HBOC syndrome have been performed with specific populations, that is, with young women diagnosed with breast cancer and/or with an investigation of specific gene regions. 37 38 Therefore, there is a need to obtain more consistent data related to HBOC syndrome from patients and their families in Brazil.

The identification of patients and family members at risk for hereditary cancer is essential, as the cumulative vital risk is much higher in affected people. This identification makes it possible to know the risk that family members are exposed to, through screening measures that allow for an early diagnosis and the implementation of appropriate follow-up and treatment protocols, which can improve the outcome of the disease in patients and their families. 39

A study conducted in the United States found that, in one year, only 8% of 603 women referred for genetic counseling, according to the NCCN criteria, attended the exam. This exemplifies how genetic counseling is still underused, even in developed countries. 11 Studies highlight the importance of providing patients better information about the genetics of cancer and the risk of hereditary cancer, as well as the role of the genetic counseling service, more specifically disease management, and therapeutic approaches that could implement greater demand for these services. 11 In studies on the assistance of specialized genetic services in Brazil, Llerena (2002) 40 and Horovitz et al. (2012), 39 highlighted the inadequate number of geneticists doctors available, the centralization of services in the private system, usually located in urban centers, and the scarcity of geneticists in the public health system, being available only in some research institutions and universities. Furthermore, the profession of genetic counselor is still not recognized in Brazil, despite the involvement of many health professionals with specialty in genetics.

As a limitation of the study, we emphasize that genetic screening was not performed in the investigated population, particularly in relation to the *BRCA* genes, which would enable a better characterization of the increased risk for hereditary breast cancer, and the associations raised. It is worth mention the high cost of the test to assess pathogenic mutations in the *BRCA1* and *BRCA2* genes, which makes its use very restricted in countries with limited sources, such as Brazil.

In this regard, information on tumor histopathology, routinely collected at the time of diagnoses, such as histological grade, the status of estrogen, progesterone, or HER-2, as well as identification of cell proliferation markers, can be incorporated into the criteria already recommended for investigation of the risk of hereditary breast and ovarian cancer, aiming to strengthen and expand the adoption of a practical strategy for genetic evaluation in patients with breast cancer. This becomes particularly important for countries in which specialized genetic services are scarce, financial resources for health are limited and concentrated in more developed regions, and where public and private health systems have major restrictions on coverage of such molecular analyzes, such as in Brazil and other Latin American countries. 12

## Conclusion

The results of this study allow the characterization of the clinical, histological, and immunohistochemical profile of women with breast cancer in a Brazilian hospital-based cohort, according to the risk of hereditary breast cancer. The findings show the possibility of considering, even if only in a complementary way, the frequency of some of these characteristics to help identify and manage women with clinical suspicion of hereditary breast cancer. In this regard, there is a higher frequency of tumors with HG3, K _i_ -67 ≥ 25%, absence of hormone receptors, and HER-2, and family history of breast cancer in women classified in this study as at increased risk for HBOC syndrome. Based on the clinical criteria routinely used in the diagnosis of breast cancer, this study collaborated to guide the care practice of genetic counseling for patients at increased risk of hereditary breast cancer in contexts such as in Brazil. Therefore, it guides the indication of women to undergo genetic evaluation and who, consequently, could benefit from these more specific therapies. It also contributes to the risk management of their families, enabling the application of specific preventive measures.
